# Clinical and cardiac characteristics of primary bilateral macronodular adrenal hyperplasia

**DOI:** 10.5937/jomb0-43319

**Published:** 2024-01-25

**Authors:** Sisi Miao, Lin Lu, Shengyong Si, Dandan Peng, Ya Zhong, Zhijing Li, Zhenqiu Yu

**Affiliations:** 1 The affiliated Hospital of Guizhou Medical University, Department of Hypertension, Guiyang, China; 2 Guizhou Medical University, Guiyang, China + Guizhou Medical University, School of Clinical Medicine, Guiyang, China; 3 Chinese Academy of Medical Science and Peking Union Medical College, Peking Union Medical College Hospital, Department of Endocrinology, Key Laboratory of Endocrinology of National Health Commission, Beijing, China

**Keywords:** primary bilateral macronodular adrenal hyperplasia, PBMAH, cardiac, echocardiogram, steroid hormone, primarna bilateralna makronodularna hiperplazija nadbubrežne žlezde, PBMA, srčana, ehokardiogram, steroidni hormon

## Abstract

**Background:**

Cardiovascular disease is the leading cause of death in Cushingžs syndrome (CS). Primary bilateral macro-nodular adrenal hyperplasia (PBMAH), is a rare cause of CS that is clinically distinct from the other common types of CS, but cardiac characteristics have been poorly studied.

**Methods:**

The clinical data, steroid hormones and echocardiographic variables of 17 patients with PBMAH were collected. Twenty-one CS patients with cortisol-producing adenoma (CPA) were collected as controls. The similarities and differences of clinical and cardiac features between the two groups were compared.

## Abstract (sr)

**Uvod:**

Kardiovaskularne bolesti su vodeći uzrok smrti kod Kušingovog sindroma (CS). Primarna bilateralna makro-nodularna hiperplazija nadbubrežne žlezde (PBMA ) je redak uzrok CS koji se klinički razlikuje od drugih uobičajenih tipova CS, ali kardijalne karakteristike su slabo proučavane.

**Metode:**

Prikupljeni su klinički podaci, steroidni hormoni i ehokardiografske varijable 17 pacijenata sa PBMA. Dvadeset i jedan pacijent sa CS sa adenomom koji proizvodi kortizol (CPA) je sakupljen kao kontrola. Upoređene su sličnosti i razlike kliničkih i kardijalnih karakteristika između dve grupe.

**Rezultati:**

U grupi PBMA udeo žena je bio manji (35,30 prema 100,00%), starosna dob je bila starija (55,76±2,42 godine prema 39,57±2,72 godine), a tok bolesti je bio duži 13,00 (5,00, 50) godine u odnosu na 1,58 (1,00, 5,00) godina. Procenat pacijenata sa PBMA sa hipertenzijom bio je veći nego kod pacijenata sa CPA (100,00% naspram 61,90%). Kortizol u plazmi i 24-časovni kortizol bez mokraće (24-časovni UFC) bili su niži kod pacijenata sa PBMA nego kod pacijenata sa CPA jutarnji kortizol 19,50 (15,35, 24,48) mg/dL naspram 28,30 (22,88, 29,89) mg/dL, 04h/dL, 04h/dL. 156,58) naspram 506,23 (292,53, 712,18) mg. Grupa PBMA imala je duži prečnik desne komore (24,06±1,23 mm naspram 20,48±0,83 mm), prečnik leve pretkomore (39,41±1,15 mm naspram 32,86±0,76 mm) i veću stopu dijastolne funkcije od 53% (s 76 ± 0,8%). CPA grupa. Univarijantna regresiona analiza je pokazala da kombinacija hipertenzije i trajanja hipertenzije ima značajne efekte na prečnik leve pretkomore (b=6,383, P=0,001; b = 0,276, P<0,001, respektivno) i E/A odnos (b=-0,496, P < 0,001, b=0,022, P<0,001, respektivno). Multivarijantna regresiona analiza je pokazala da nivo kortikosterona ima značajan uticaj na sistolni krvni pritisak (b=6,712, P=0,025).

**Zaključak:**

U poređenju sa CPA, ventrikularna dijastolna disfunkcija je česta kod pacijenata sa PBMA pod relativno nižim nivoom kortizola. PBMA je imao visok nivo kortikosterona koji može igrati ulogu u razvoju hipertenzije i daljih srčanih promena.

## Introduction

Cushing’s syndrome (CS), or endogenous hypercortisolism, is a kind of serious endocrine disease characterized by chronic, autonomous and excessive secretion of cortisol due to a variety of causes. It is estimated that the prevalence of CS is about 40 cases per million and the incidence is about 0.7–2.4 cases per million per year [1] [2] [3]. Although rare, CS is related to higher mortality. A meta-analysis showed that pituitary-dependent CS, also known as Cushing’s disease, had a standardized mortality rate (SMR) of 1.84 (95% CI 1.28–2.65) [4]. SMR for adrenal-dependent CS varied widely among studies, ranging from 1.35 to 7.5 for adrenal adenomas [5] [6] [7] [8] [9], 1.14–12 for bilateral adrenal hyperplasia [5] [7] [8], and as high as 48 (95% CI 30.75–71.42) for adrenal cortical carcinoma [8] [9]. Cardiovascular disease is the leading cause of death in CS. Hazard ratio of myocardial infarction in CS was 2.1 (95%CI 0.5-8.6), and that of the heart failure was 6.0 (95% CI 2.1–17.1) [10]. CS patients are prone to left ventricular hypertrophy and diastolic dysfunction [11] [12] [13], and left ventricular hypertrophy is more obvious than that in the hypertensive control group [11].

Primary bilateral macronodular adrenal hyperplasia (PBMAH) is a rare cause of CS, accounting for less than 2% of CS [14]. Most PBMAH progress slowly and have no obvious clinical manifestations, or present mildly; a small number of patients have obvious centripetal obesity, purple lines and/or other obvious symptoms of hypercortisolism. Cortisol levels in PBMAH patients range from normal to significantly elevated and the adrenal steroid hormone spectrum was different in the PBMAH patients from the CS caused by cortisol producing adenoma (CPA) [15]. PBMAH is characterized by multiple nodules > 1 cm in both adrenal glands, with adrenal volume up to 4-24 times the normal adrenal volume. Although, the clinical manifestations are not obvious, comorbidities in PBMAH patients are very common. In a study involving 102 PBMAH patients form China, PBMAH had higher rates of hypertension and diabetes than CPA (83.3% vs. 61.8%, 43.1% vs. 26.6%, respectively), while the rates of dyslipidemia were roughly similar (37.3% vs. 24.9%, P > 0.05) [16]. In another study from France involving 98 PBMAH patients, the proportion of hypertension (69.3%), diabetes (33.7%) and dyslipidemia (12.9%) were also not low [17]. PBMAH differs significantly from the other common types of CS, but the resulting changes in cardiac structure and function have been poorly characterized.

In this study, clinical information and echocardiographic variables were collected to investigate cardiac changes in PBMAH patients and CS caused by CPA patients.

## Materials and methods

### Subjects

We conducted a retrospective study using a hospital information system to collect clinical data from patients diagnosed with PBMAH who underwent echocardiography during the last 3 years (October 1, 2019 to September 30, 2022) at Peking Union Medical College Hospital. For comparison, patients with CS caused by CPA were selected as controls.

No personal information of patients would be disclosed in this study, so informed consent was not required.

### Inclusion/Exclusion criteria

Patients were diagnosed with CS when they had typical clinical manifestations, such as central obesity, thin skin, purple striae and meet 2 or more of the following criteria: (1) Elevated levels of 24 h urine free cortisol (24 h UFC) above the upper limit of the reference interval; (2) Midnight serum cortisol levels > 1.8 mg/dL; (3) 1 mg overnight dexamethasone suppression test (DST) or 2 mg classic low dose dexamethasone suppression test were not suppressed (early morning serum cortisol > 1.8 μg/dL or 24 h UFC was not suppressed below the lower limit of normal range after administration).

These clinical manifestations and hormonal characteristics were present or absent in PBMAH patients, while adrenal imaging in all PBMAH patients showed bilateral large nodules (≥1 cm) with internodal hyperplasia and/or atrophy. All the CPA patients met the above diagnostic criteria for CS, and the adrenal imaging suggested adrenal adenomas and atrophy of the contralateral adrenal.

The exclusion criteria were as follows: (1) Congenital cardiovascular disease; (2) Valvular heart disease; (3) Amyloid cardiomyopathy, (4) Any cardiomyopathies diagnosed before the onset of CS symptoms; (5) Systemic autoimmune disease; (6) Uncontrolled thyroid disease; (7) Recent infection, or acute illness; (8) Long-term using glucocorticoids, estrogen (>3 months); (9) Pregnancy.

We did not exclude coronary heart disease, which is usually one of the adverse consequences of hypercortisolism. The Ethics Committee of Peking Union Medical College Hospital approved this study.

### Echocardiogram

All patients underwent standard M-mode echocardiography and tissue Doppler imaging. Left ventricular hypertrophy was defined as left ventricular mass index ≥ 115 g/m^2^ in males or ≥ 95 g/m^2^ in females. Interventricular septal diameter (IVSd) or left ventricle posterior wall diameter (LVPWd) > 11 mm in men or > 10.5 mm in females were the reference indexes for left ventricular hypertrophy. Left ventricular ejection fraction (LVEF) < 52% in men or < 53% in women indicates abnormal left ventricular systolic function. Equal to or more than 2 of the following variables indicated abnormal left ventricular diastolic function: the average E/e’ ratio of the peak E velocity to the lateral and interstitial motion velocity (e’) of the mitral annulus in the early stage of horizontal mitral diastole was more than 14; the mitral annulus ventricular septal e’ velocity < 7 cm/s, or the lateral wall e’ < 10 cm/s; left atrial volume index > 34 mL/m^2^; the maximum regurgitation velocity of tricuspid valve was more than 2.8 m/s.

### Steroid hormones

High-performance liquid chromatography-tandem mass spectrometry (LC-MS/MS) was used to detect plasma steroid hormones. The mass spectrometry analysis platform was Waters TQS liquid chromatography tandem mass spectrometer (America). Waters ACQUITY UPLC BEH C8 column (1.7 μm, 2.1×100 mm) were used for liquid chromatography separation. The pretreatment process was to add methanol to precipitate the protein impurities in the serum; The steroid hormones were extracted by using Oasis PRiME HLB extraction board, and then dried under nitrogen; Finally, the steroid hormones were extracted by acetonitrile and n-hexane, mixed, centrifuged, and tested by machine.

### Statistical analysis

Qualitative data are expressed in frequency and percentage terms. If the quantitative data conform to normal distribution, it is expressed as mean ± standard deviation. If it does not fit the normal distribution, it is expressed as the median (25th percentile, 75th percentile). Independent two-sample t-test, Chi-square test, and nonparametric rank-sum test of two independent samples were used to evaluate statistical significance. Linear regression analysis (univariate and multivariate) was used to investigate the correlation between variables and cardiac variables and blood pressure levels P <0.05 indicates statistical difference. Statistical analysis was performed using SPSS version 25.0 software (IBM Corp., Armonk, NY, USA).

## Results

### Clinical characteristics

Total of 38 cases (17 PBMAH and 21 CPA cases) were included in this study. At the time of first definitive diagnosis, the age of PBMAH patients were older than CPA patients (55.76 ± 2.42 years vs. 39.57 ± 2.72 years). PBMAH group showed a lower proportion of women (35.3%) than that of CPA group (100%). There was no significant difference in waist circumference, but the PBMAH group had a higher body mass index (28.36 ± 1.28 kg/m^2^ vs. 25.49 ±0.74 kg/m^2^). Both proportion of smoking and drinking history were higher in PBMAH group (Table 1).

**Table 1:** Clinical characteristics of PBMAH and CPA patients. BMI: body mass index. MI: myocardial infarction. PCI: percutaneous coronary intervention. SBP: systolic blood pressure, DBP: diastolic blood pressure. ACEI: angiotensin-convert enzyme inhibitors, ARB: angiotensin receptor blockers. CCB: calcium channel blockers.

	PBMAH Group<br>(n=17)	CPA Group<br>(n=21)	P value
Sex (female)	6/17 (35.30)	21/21 (100.00)	< 0.001
Age (years)	55.76 ± 2.42	39.57 ± 2.72	< 0.001
BMI (kg/m^2^)	28.36 ± 1.28<br>(n = 13)	25.49 ± 0.74<br>(n = 18)	0.048
Waistline (cm)	98.89 ± 4.69	94.29 ± 2.86	0.383
Smoking			
Never	6/17 (35.30)	21/21 (100.00)	
Quited	4/17 (23.50)	0/21 (0.00)	< 0.001
Smoking	7/17 (41.20)	0/21 (0.00)	
Drinking			
Never	8/17 (47.10)	21/21 (100.00)	
Quited	6/17 (35.30)	0/21 (0.00)	< 0.001
Drinking alcohol	3/17 (17.60)	0/21 (0.00)	
Course of CS	13.00 (5.00, 21.50)	1.58 (1.00, 5.00)	< 0.001
Coronary artery disease	2/17 (11.80)	0/21 (0.00)	0.193
MI	2/17 (11.80)	0/21 (0.00)	0.193
PCI	2/17 (11.80)	0/21 (0.00)	0.193
Hypertension	17/17 (100.00)	13/21 (61.90)	0.005
Duration of hypertension (years)	14.00 (5.00, 21.5)	(0.00, 1.00)	< 0.001
Highest SBP (mmHg)	± 5.12	170.67 ± 6.24	0.555
Highest DBP (mmHg)	107.50 ± 3.06<br>(n = 16)	111.92 ± 3.45<br>(n = 12)	0.349
Number of antihypertensive drugs	1.88 ± 0.32	0.76 ± 0.26	0.002
ACEI/ARB	8/17 (47.10)	0/21 (0.00)	< 0.001
β blockers	6/17 (35.30)	2/21 (9.50)	0.107
Dihydropyridine CCB	12/17 (70.60)	8/21 (38.10)	0.058
Diuretic (antisterone not included)	1/17 (5.90)	2/21 (9.50)	>0.999
Antisterone	3/17 (17.60)	1/21 (4.80)	0.307
α blockers	1/17 (5.90)	2/21 (9.5)	>0.999
SBP this visit (mmHg)	140.29 ± 4.98	139.95 ± 3.71	0.956
DBP this visit (mmHg)	89.82 ± 4.03	90.19 ± 3.46	0.945
Dyslipidemia	13/17 (81.30)	20/21 (95.20)	0.296
Glycomotablism			
Euglycemia	3/17 (17.60)	8/21 (38.10)	
Impaired glucose tolerance	6/17 (35.30)	5/21 (23.80)	0.496
Diabetes	8/17 (47.10)	8/21 (38.10)	
Duration of pathoglycemia (years)	1.00 (0.00, 5.50)<br>(n = 17)	0.00 (0.00, 0.00)<br>(n = 21)	0.003

The time from onset of some symptoms to diagnosis was longer in the PBMAH group than in theCPA group [13.00 (5.00, 21.50) years vs. 1.58 (1.00, 5.00) years]. The PBMAH group also had a higher proportion of combining with hypertension (100.00% vs. 61.90%), a longer duration of hypertension [14.00 (5.00, 21.5) years vs. 0.0 (0.00, 1.00) years], a wider variety of antihypertensive drugs [1.88 ± 0.32 kinds vs. 0.76 ± 0.26 kinds]. There were no significant differences in the proportion of abnormal glucose metabolism and dyslipidemia between the two groups (Table 1).

### Biochemical variables

PBMAH group had lower total cholesterol (TC, 5.42 ± 0.41 mmol/L vs. 6.57 ± 0.33 mmol/L), low-density lipoprotein cholesterol (LDL-C, 3.40 ± 0.27 mmol/L vs. 4.25 ± 0.27 mmol/L), and high-density lipoprotein cholesterol [(HDL-C, 1.20 (1.03, 1.40) mmol/L vs 1.59 (1.34, 1.72) mmol/L] levels, while had higher fasting blood glucose (10.82 ± 0.84 mmol/L vs 10.27 ± 1.16 mmol/L), glycosylated hemoglobin [6.10 (5.70, 6.60) % vs. 5.50 (5.20, 5.90) %], and HOMA-index (4.64 ± 0.57 vs. 2.94 ± 0.37). Free triiodothyronine [FT3, 3.31 (2.83, 3.68) ng/L vs. 2.34 (1.97, 2.68) ng/L] and free thyroxine [FT4, 1.23 (1.11, 1.43) ng/dL vs. 1.04 (0.96, 1.27) ng/dl] were higher in PBMAH group, and thyroid stimulating hormone (TSH) showed a higher value although there was no statistical difference [1.50 (0.82, 1.94) mIU/L vs. 1.13 (0.55, 1.70) mIU/L]. The PBMAH group had a higher creatinine level (80.94 ± 3.22 μmol/L vs. 66.95 ± 3.02 μmol/L). The blood sodium level in PBMAH group was lower than that in CPA group [139.50 (138.25, 140.00) mmol/L vs. 141.00 (140.00, 142.50) mmol/L], while there was no significant difference in blood potassium, 24 h urinary potassium and sodium (Table 2).

**Table 2:** Biochemical variables of PBMAH and CPA patients. TC: total cholesterol, TG: triglyceride, LDL-C: low-density lipoprotein cholesterol, HDL-C: high-density lipoprotein cholesterol. HOMA: homeostasis model assessment. FT3: Free triiodothyronine, FT4: Free thyroxine, TSH: Thyroid stimulating hormone. ALT: Alanine aminotransferase, AST: Aspartate aminotransferase. ACR: albumin-to-creatinine ratio. T-25OHD: total 25-hydroxyvitamin D, PTH: parathyroid hormone. GH: growth factor, IGF1: insulin-like growth factor 1. ACTH: Adrenocorticotropic Hormone, UFC: Urinary free cortisol, LDDST: Low-dose dexamethasone suppression test, HDST: High-dose dexamethasone suppression test.

	PBMAH Group<br>(n=17)	CPA Group<br>(n=21)	P Value
TC (mmol/L)	5.42 ± 0.41	6.57 ± 0.33	0.033
TG (mmol/L)	1.37 (0.98, 2.29)	1.18 (0.93, 2.13)	0.592
LDL-C (mmol/L)	3.40 ± 0.27	4.25 ± 0.27	0.037
HDL-C (mmol/L)	1.20 (1.03, 1.40)	1.59 (1.34, 1.72)	0.010
FBG (mmol/L)	7.50 (6.23, 8.65)	5.30 (4.65, 6.85)	0.002
2-h BG (mmol/L)	10.82 ± 0.84<br>(n = 11)	10.27 ± 1.16<br>(n = 18)	0.707
Fasting insulin (mIU/L)	14.36 ± 1.75<br>(n = 10)	12.01 ± 1.52<br>(n = 12)	0.320
2-h insulin (mIU/L)	115.44 (33.55, 188.83)<br>(n = 10)	93.77 (42.94, 179.18)<br>(n = 12)	>0.999
Glycated hemoglobin (%)	6.10 (5.70, 6.60)<br>(n = 13)	5.50 (5.20, 5.90)<br>(n = 19)	0.017
HOMA-index	4.64 ± 0.57<br>(n = 10)	2.94 ± 0.37<br>(n = 12)	0.018
FT3 (ng/L)	3.31 (2.83, 3.68)	2.34 (1.97, 2.68)	0.002
FT4 (ng/dL)	1.23 (1.11, 1.43)	1.04 (0.96, 1.27)	0.049
TSH (mIU/L)	1.50 (0.82, 1.94)	1.13 (0.55, 1.70)<br>(n = 18)	0.150
ALT (U/L)	20.00 (13.50, 36.75)	24.00 (18.50, 33.50)	0.560
AST (U/L)	20.00 (15.00, 22.00)	17.50 (16.00, 22.50)	0.483
Creatinine (mmol/L)	80.94 ± 3.22	66.95 ± 3.02	0.003
Uric Acid (mmol/L)	410.31 ± 38.66	357.95 ± 28.54	0.272
Urinary ACR (mg/g creatinine)	9.00 (6.00, 20.00)<br>(n = 11)	19.00 (10.75, 41.00)<br>(n = 10)	0.148
Minimum serum potassium (mmol/L)	3.47 ± 0.18	3.55 ± 0.09	0.667
Minimum Serum sodium (mmol/L)	139.50 (138.25, 140.00)	141.00 (140.00, 142.50)	0.019
24h urinary potassium (mmol/24h)	48.00 (43.80, 66.20)<br>(n = 11)	46.60 (24.90, 73.25)<br>(n = 5)	0.533
24h urinary sodium<br>(mmol/24h)	170.00 (131.00, 230.00)<br>(n = 11)	117.50 (80.50, 182.25)<br>(n = 4)	0.192
T-25OHD (μg/L)	15.40 (12.30, 22.00)<br>(n = 11)	14.60 (10.30, 19.00)<br>(n = 14)	0.701
PTH (ng/L)	47.28 ± 5.16<br>(n =10)	64.51 ± 4.81<br>(n = 14)	0.025
GH (μg/L)	0.20 (0.10, 0.98)	0.40 (0.20, 1.50)	0.208
IGF1 (μg/L)	178.43 ± 21.15	212. 71 ± 18.59	0.298
ACTH < minimum measurable value	4/17 (23.50)	18/21 (85.70)	<0.001
Baseline 24h UFC (μg/24h)	106.20 (65.35, 156.58)<br>(n =16)	506.23 (292.53, 712.18)	<0.001
LDDST 24h UFC (μg/24h)	61.90 (37.40, 134.00)<br>(n = 7)	347.40 (167.90, 608.50)<br>(n = 12)	0.007
HDDST 24h UFC (μg/24h)	217.23 ± 152.95<br>(n = 3)	492.22 ± 59.71<br>(n = 13)	0.075
Cortisol 8 am (μg/dL)	19.50 (15.35, 24.48)	28.30 (22.88, 29.89)	0.002
Cortisol 4 pm (μg/dL)	7.31 (3.54, 9.60)<br>(n = 5)	24.65 (13.01, 28.73)<br>(n = 6)	0.006
Cortisol 0 am (μg/dL)	6.91 ± 1.26<br>(n =10)	23.12 ± 1.83<br>(n = 13)	<0.001
LDDST Cortisol (μg/dL)	3.60 (2.10, 8.60)<br>(n = 15)	25.90 (21.16, 29.70)<br>(n = 15)	<0.001
HDDST Cortisol (μg/dL)	12.19 (6.60, 32.26)<br>(n = 5)	29.00 (24.19, 31.05)<br>(n = 9)	0.205
Renin activity (μg/L/h)	0.41 (0.01, 1.08)<br>(n = 13)	0.38 (0.20, 1.09)<br>(n =12)	0.326
Angiotensin (ng/L)	59.04 (44.77, 97.72)<br>(n =14)	62.24 (52.54, 73.05)<br>(n =12)	0.681
Aldosterone (ng/dL)	14.39 (10.55, 16.99)<br>(n = 14)	17.36 (15.30, 22.74)<br>(n =12)	0.016
3-methoxy-norepinephrine (nmol/L)	0.45 (0.36, 0.47)<br>(n = 11)	0.21 (0.12, 0.29)<br>(n = 11)	0.006
3-methoxy-epinephrine (nmol/L)	0.09 (0.04, 0.16)<br>(n = 11)	0.07 (0.06, 0.12)<br>(n = 11)	0.355
3-methoxy-tyramine (nmol/L)	0.02 (0.01, 0.03)<br>(n = 5)	0.02 (0.01, 0.04)<br>(n = 4)	0.806
24h urinary epinephrine (mg/24h)	39.09 (16.50, 46.95)<br>(n = 10)	24.10 (13.30, 34.95)<br>(n = 13)	0.107
24h urinary norepinephrine (mg/24h)	2.58 (1.29, 5.10)<br>(n = 10)	2.33 (0.80, 3.80)<br>(n = 13)	0.385
24h urinary dopamine (mg/24h)	223.47 (113.98, 314.58)<br>(n = 10)	196.50 (91.00, 270.50)<br>(n = 13)	0.457

### Hormone variables

The proportion of ACTH below the lowest measurable range was significantly lower in the PBMAH group than that in the CPA group (23.5% vs 85.7%). 24h UFC, at baseline and after low-dose dexamethasone suppression test (LDDST), were lower in the PBMAH group than that in the CPA group. Plasma cortisol (8 am, 0 am, 4 pm and after LDDST) were lower in PBMAH group, too. Although there was no statistical difference in 24-h UFC and plasma cortisol after high-dose dexamethasone suppression test (HDDST) between the two groups, the PBMAH group showed a lower value (Table 2).

The aldosterone level in PBMAH group was lower than that in CPA group [14.39 (10.55, 16.99) μg/L vs. 17.36 (15.30, 22.74) μg/L], while renin activity [0.41 (0.01, 1.08) μg/L/h vs. 0.38 (0.20, 1.09) μg/L/h] and angiotensin II [59.04 (44.77, 97.72) ng/L vs. 62.24 (52.54, 73.05) ng/L] levels were not significantly different in the 2 groups. Diagnosis of pheochromocytoma or paraganglioma was not considered in all patients, but the PBMAH group had a higher 3-methoxy-epinephrine than that in the CPA group [0.45 (0.36, 0.47) nmol/L vs. 0.21 (0.12, 0.29) nmol/L], and a higher trend in 24 h urinary epinephrine (Table 2).

Plasma steroid hormones were detected in 10 PBMAH patients and 4 CPA patients by LC-MS/MS. Due to the small number of cases included and some variables below the minimum measurable range, we show the proportion of these data below the measurable range in the 2 groups (Table 3). The proportion of 21-deoxycortisol below the measurable range was significantly higher in the CPA group than in the PBMAH group (20% vs. 100%).

**Table 3:** Steroid hormones detected by LC-MS/MS of PBMAH and CPA patients DHEA: dehydroepiandrosterone, DHEA-S: dehydroepiandrosterone sulfate

	PBMAH Group (n=10)	CPA Group (n=4)	P Value
Pregenolone < minimum measurable value	7/10 (70.0)	4/4 (100.0)	0.505
Progesterone < minimum measurable value	3/10 (30.0)	3/4 (75.0)	0.245
11-deoxycortone	0.08 (0.05, 0.30)	0.06 (0.06, 0.07)	0.436
Corticosterone	4.04 (0.92, 15.83)	2.08 (1.38, 3.74)	0.671
18-hydroxycorticosterone	0.57 (0.22, 1.14)<br>(n = 9)	0.43 (0.40, 0.71)	0.938
Aldosterone < minimum measurable value	3/10 (30.0)	2/4 (50.0)	0.580
17-hydroxypregnenolone	0.52 (0.34, 0.82)	0.15 (0.08, 0.18)	0.007
17-hydroxyprogesterone	0.87 (0.39, 1.21)	0.20 (0.11, 0.62)	0.034
11-deoxycortisol	0.67 (0.39, 1.13)	0.85 (0.58, 1.32)	0.396
21-deoxycortisol < minimum measurable value	2/10 (20.0)	4/4 (100.0)	0.015
18-oxycortisol < minimum measurable value	6/9 (66.7)	2/4 (50.0)	>0.999
18-hydroxycortisol	1.19 (0.62, 2.27)<br>(n = 9)	0.91 (0.70, 1.17)	0.537
DHEA < minimum measurable value	4/10 (40.0)	4/4 (100.0)	0.085
DHEA-S	470.00 (177.00, 876.75)	107.50 (70.50, 248.00)	0.090
Androstendinoe	0.53 (0.29, 0.69)	0.29 (0.26, 0.61)	0.396
11-ketone-testosterone	0.21 (0.14, 0.35)	0.20 (0.12, 0.28)	0.395
11 -hydroxytestosterone	0.13 (0.08, 0.33)	0.10 (0.08, 0.18)	0.671

**Figure 1:**
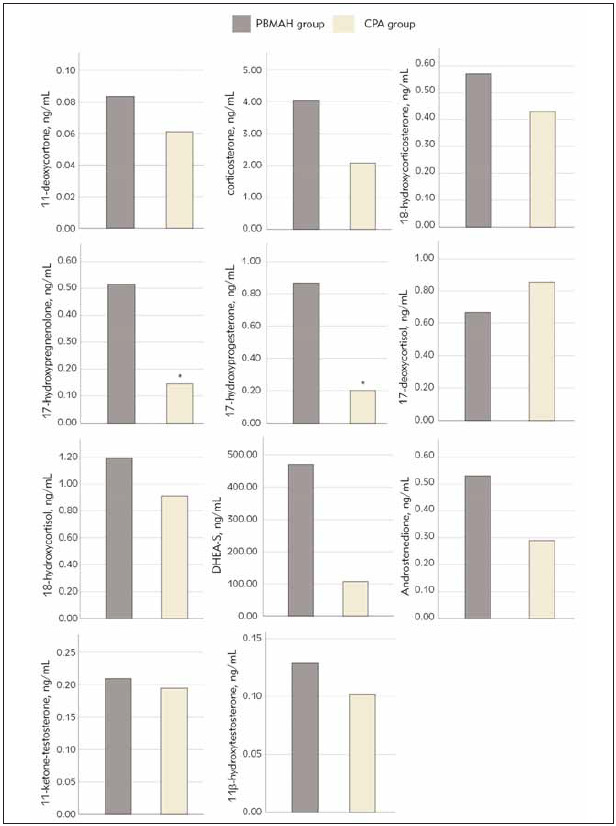
Steroid hormones of PBMAH and CPA group. *Different from PBMAH group.

Of those measures that could be measured, the PBMAH group had significantly higher levels of 17-hydroxyprogesterone and 17-hydroxyprogesterone than that in the CPA group. The concentrations of 11-deoxycorticosterone, corticosterone, 18-hydroxycorticosterone, 18-hydroxycortisol, dehydroepiandrosterone sulfate (DHEA-S), androstenedione and 11-hydroxytestosterone in the PBMAH group all showed higher level without significant differences, while the 11-deoxycortisol showed a lower lever (supplementary Table 3 and Figure 1).

### Echocardiographic variables

Right ventricular diameter (24.06 ± 1.23 mm vs. 20.48 ± 0.83 mm) and left atrial diameter (39.41 ± 1.15 mm vs. 32.86 ± 0.76 mm) were larger in the PBMAH group than that of CPA group. The left ventricular mass index and the left ventricular end-diastolic volume index in PBMAH group showed a greater trend (85.06± 6.12 g/m^2^ vs. 74.36±5.50 g/m^2^, 53.40±2.25 mL/m^2^ vs. 48.95 ± 3.06 ml/m^2^,respectively), without significant differences. There were no patients with decreased systolic function in either group. LVEF and left ventricular fraction shortening (LVFS) in the PBMAH group were higher (median 68% vs. 65%, 39% vs. 36%, respectively). There was no significant difference in the ratio of combining with left ventricular hypertrophy (17.6% vs. 38.1%), but the ratio of combining with left ventricular diastolic dysfunction was higher in the PBMAH group than that of the CPA group (76.5% vs. 38.1%) (Table 4).

**Table 4:** Echocardiographic variables of PBMAH and CPA patients. RV: right ventricle, LA: left atrium, LVED: left ventricle end-diastolic IVSd: interventricular septum diameter, LVPWd: left ventricular posterior wall diameter, LV: left ventricle, LVEF: left ventricular ejection fraciont, LVFS: left ventricular fractional shortening, E: early wave transmitral diastolic velocity, A: late wave transmitral diastolic velocity, E’: early diastolic velocity of septal and lateral myocardial portions at tissue-Doppler imaging.

	PBMAH Group<br>(n=17)	CPA Group<br>(n=21)	P Value
RV diameter (mm)	24.06 ± 1.23	20.48 ± 0.83	0.017
LA diameter (mm)	39.41 ± 1.15	32.86 ± 0.76	<0.001
LVED diameter (mm)	46.65 ± 0.82	44.19 ± 1.14	0.102
IVSd (mm)	10.00 (8.00, 10.50)	9.00 (8.00, 11.00)	0.622
LVPWd (mm)	9.35 ± 0.37	9.10 ± 0.36	0.624
Relative wall thickness	0.41 ± 0.01	0.42 ± 0.02	0.608
LV mass (g)	155.87 ± 10.51	136.57 ± 9.05	0.171
LV mass index (g/m^2^)	85.06 ± 6.12<br>(n = 13)	74.36 ± 5.50<br>(n = 18)	0.208
LVED volume (mL)	101.30 ± 4.09	90.31 ± 5.25	0.120
LVED volume index (mL/m^2^)	53.40 ± 2.25	48.95 ± 3.06	0.285
LV hypertrophy	3/17 (17.6)	8/21 (38.1)	0.282
LVEF (%)	68.00 (66.00, 73.50)	65.00 (59.50, 69.00)	0.024
LVFS (%)	39.00 (36.50, 43.50)	36.00 (32.00, 39.00)	0.007
E/A ratio	0.60 (0.60, 0.90)	1.05 (0.73, 1.28)	0.006
E/e’ ratio	8.57 ± 1.19<br>(n = 7)	8.00 ± 0.85<br>(n = 8)	0.697
Tricuspid regurgitation velocity (m/s^2^)	2.25 ± 0.08	2.11 ± 0.07	0.177
Diastolic dysfunction	13/17 (76.5)	8/21 (38.1)	0.025

### Analysis of correlation

Left ventricular mass index (LVMD), relative wall thickness (RWT), left atrial (LA) diameter, E/A ratios and E/e’ ratios were used as dependent variables to conduct univariate regression analysis. Some variables that have significant influence on LVMD included duration of hypertension, systolic blood pressure (max), cortisol 4 pm and 24 h urinary dopamine. That have significant influence on RWT included waistline, parathyroid hormones, cortisol 4 pm, 24 h urinary dopamine and progesterone. Many variables, such as gender, age, smoking history, drinking alcohol history, course of CS, combining with coronary artery disease, combining with hypertension, duration of hypertension, diastolic blood pressure, numbers of used antihypertensive drugs, baseline 24 h UFC, Cortisol 8 am, Cortisol 0 am, LDDST cortisol and so on, had significantly influences on LA diameter. There were also many variables that had significantly influence on E/A ratios, such as gender, age, smoking history, course of CS, combining with hypertension, numbers of used antihypertensive drugs, baseline 24 h UFC, LDDST 24 h UFC, cortisol 8 am, LDDST cortisol, progesterone and 17-hydroxypregnenolone. Creatinine and urinary albumin/creatinine ratio had significantly influence on E/e’ ratios (Table 5). We intended to conduct multivariate regression analysis to explore the factors affecting cardiac damage, but due to too many correlation indicators and limited sample size, it was difficult to construct an effective multivariate regression model.

**Table 5:** Univariate linear regression analysis of echocardiographic indicators as dependent variables in PBMAH and CPA patients. LV: left ventricular, LA: left atrial, RWT: relative wall thickness, E, early wave transmitral diastolic velocity; A, late-wave transmitral diastolic velocity; e’, early diastolic velocity of septal and lateral myocardial portions at tissue-Doppler imaging. BMI: body mass index. CS: Cushing’s syndrome, CAD: coronary artery disease. ACEI: angiotensin-convert enzyme inhibitors, ARB: angiotensin receptor blockers. CCB: calcium channel blockers. TC: total cholesterol, TG: triglyceride, LDL-C: low-density lipoprotein cholesterol, HDL-C: high-density lipoprotein cholesterol. FBG: fasting blood glucose, BG: blood glucose, HOMA: homeostasis model assessment. FT3: Free triiodothyronine, FT4: Free thyroxine, TSH: thyroid stimulatinghormone. ACR: albumin-to-creatinine ratio. T-25OHD: total 25-hydroxyvitamin D, PTH: parathyroid hormone. GH: growth factor, IGF1: insulinlike growth factor 1. UFC: Urinary free cortisol, LDDST: Low-dose dexamethasone suppression test, HDDST: High-dose dexamethasone suppression test. DHEA: dehydroepiandrosterone, DHEA-S: dehydroepiandrosterone sulfate.

Variables	LV Mass Index		RWT		LA diameter		E/A ratios		E/e’ ratios	
	b	P value	b	P value	b	P value	b	P value	r	P value
Gender	15.255	0.095	0.008	0.786	7.717	<0.001	-0.278	0.020	1.400	0.360
Age	0.504	0.084	<-0.001	0.917	0.185	0.002	-0.018	<0.001	0.055	0.342
BMI	0.183	0.864	0.000	0.940	0.455	0.074	-0.023	0.146	-0.079	0.665
Waistline	0.750	0.101	0.003	0.033	0.181	0.066	-0.013	0.025	0.049	0.456
Smoking	9.168	0.063	0.013	0.429	4.592	<0.001	-0.170	0.013	0.911	0.307
Drinking	4.428	0.496	0.003	0.888	5.174	<0.001	-0.095	0.300	0.607	0.611
Course of CS	0.652	0.133	0.001	0.344	0.273	0.001	-0.019	0.001	0.025	0.707
Combined CAD	7.636	0.658	0.018	0.758	8.139	0.030	-0.303	0.224	0.269	0.901
Combined with hypertension	18.213	0.065	-0.004	0.901	6.383	0.001	-0.496	<0.001	2.615	0.211
Duration of hypertension	0.932	0.021	0.001	0.409	0.276	<0.001	-0.022	<0.001	0.016	0.805
Systolic blood pressure	0.531	0.043	0.000	0.449	0.008	0.857	-0.004	0.113	0.014	0.742
Diastolic blood pressure	-0.050	0.904	0.001	0.273	-0.151	0.040	0.002	0.617	-0.108	0.113
Number of antihypertensive drugs	5.622	0.053	-0.002	0.876	1.783	0.004	-0.124	0.002	0.280	0.567
ACEI/ARB	4.547	0.654	-0.014	0.669	6.283	0.001	-0.094	0.494	1.333	0.462
b blockers	7.599	0.431	-0.023	0.465	5.017	0.014	-0.190	0.164	-1.045	0.525
CCB	15.756	0.056	0.004	0.880	3.611	0.031	-0.373	<0.001	1.900	0.207
Diuretic (antisterone not included)	18.996	0.267	0.024	0.619	0.590	0.854	-0.275	0.181	2.577	0.218
Antisterone	16.168	0.255	-0.009	0.828	1.353	0.630	-0.293	0.103	0.269	0.901
a blockers	37.057	0.115	-0.034	0.472	0.229	0.943	-0.203	0.328	0.269	0.901
Combined with dyslipidemia	-19.858	0.160	-0.006	0.894	-4.727	0.092	0.022	0.906	-	-
TC	-1.276	0.608	0.012	0.143	-1.460	0.005	0.004	0.906	0.238	0.671
TG	3.060	0.375	0.021	0.057	0.410	0.598	-0.066	0.181	0.769	0.462
LDL-C	-1.070	0.744	0.020	0.052	-1.785	0.010	-0.008	0.857	0.388	0.575
HDL-C	-16.975	0.159	-0.056	0.121	-7.343	0.002	-0.306	0.053	-0.559	0.777
Combined with pathoglycemia	4.509	0.398	-0.006	0.711	2.280	0.023	-0.123	0.066	-0.342	0.739
Duration of pathoglycemia	0.194	0.750	0.001	0.540	0.357	0.005	-0.013	0.150	0.010	0.901
FBG	0.848	0.675	0.003	0.687	1.327	0.001	-0.069	0.018	0.128	0.736
2h BG	0.969	0.406	0.005	0.192	0.407	0.101	-0.043	0.014	0.123	0.684
Fasting insulin	-0.531	0.594	-0.004	0.188	0.126	0.611	-0.005	0.706	-0.161	0.345
2h insulin	-0.091	0.066	0.000	0.073	-0.013	0.292	0.000	0.559	-0.010	0.153
Glycosylated hemoglobin	5.033	0.328	0.024	0.163	3.375	0.004	-0.185	0.016	1.091	0.220
HOMA-index	0.977	0.754	-0.008	0.398	1.654	0.023	-0.091	0.029	-0.066	0.904
FT3	1.580	0.831	-0.003	0.891	3.178	0.046	-0.187	0.080	-0.209	0.871
FT4	-4.298	0.848	-0.008	0.913	3.941	0.412	-0.245	0.446	0.647	0.858
TSH	-5.052	0.389	0.015	0.421	0.001	0.999	-0.060	0.504	-0.941	0.529
ACR	0.128	0.096	0.000	0.381	0.033	0.059	-0.001	0.319	0.039	0.004
Creatinine	0.529	0.053	0.000	0.865	0.156	0.006	-0.005	0.206	0.080	0.048
Uric acid	0.044	0.119	0.000	0.148	0.007	0.255	-0.001	0.004	0.000	0.958
GH	-9.936	0.102	-0.007	0.801	-1.245	0.374	0.113	0.296	-0.478	0.705
IGF-1	-0.093	0.208	0.000	0.661	-0.026	0.135	0.002	0.033	-0.017	0.201
T-25OHD	-0.447	0.640	0.001	0.576	0.000	0.997	-0.004	0.517	0.077	0.126
PTH	0.017	0.950	0.002	0.015	-0.166	0.010	0.005	0.210	-0.024	0.719
Baseline 24h UFC	-0.019	0.173	<0.001	0.800	-0.007	0.015	0.001	<0.001	0.000	0.834
LDDST 24h UFC	-0.004	0.872	<0.001	0.953	-0.007	0.175	0.001	0.003	0.006	0.227
HDDST 24h UFC	0.009	0.781	<0.001	0.800	-0.002	0.752	0.001	0.253	0.004	0.593
Cortisol 8 am	-0.393	0.484	-0.001	0.428	-0.235	0.047	0.016	0.038	-0.001	0.989
Cortisol 4 pm	1.335	0.027	0.004	0.028	-0.242	0.106	-0.009	0.395	-0.056	0.215
Cortisol 0 am	-0.023	0.965	0.001	0.747	-0.338	0.001	0.012	0.107	-0.005	0.900
LDDST Cortisol	0.113	0.726	0.001	0.581	-0.151	0.026	0.008	0.048	0.011	0.830
HDDST Cortisol	-0.061	0.948	-0.001	0.719	-0.055	0.722	0.006	0.513	0.160	0.535
Renin activity	-10.091	0.156	-0.017	0.162	-0.515	0.552	0.009	0.882	0.172	0.685
Angiotensin	-0.069	0.537	<-0.001	0.922	0.019	0.488	-0.001	0.775	0.000	0.989
Aldosterone	-0.635	0.516	-0.004	0.161	-0.156	0.491	0.005	0.765	0.048	0.660
3-methoxy-norepinephrine	-22.320	0.570	-0.019	0.760	1.665	0.750	-0.292	0.325	-0.555	0.886
3-methoxy-epinephrine	0.398	0.996	0.245	0.216	10.933	0.523	-1.552	0.105	-1.516	0.876
3-methoxy-tyramine	33.159	0.975	-0.207	0.906	-16.616	0.934	-2.377	0.721	-48.313	0.522
24h urinary norepinephrine	-0.392	0.361	0.000	0.906	0.043	0.614	-0.005	0.336	-0.026	0.675
24h urinary epinephrine	-1.553	0.561	0.002	0.778	0.585	0.274	-0.009	0.785	-0.198	0.557
24h urinary dopamine	-0.124	0.050	0.000	0.032	-0.008	0.534	0.001	0.294	-0.005	0.605
Progesterone	-11.283	0.052	-0.037	0.017	-2.554	0.058	0.216	0.007	-0.757	0.232
11-deoxycortone	21.895	0.638	0.053	0.509	-3.381	0.687	-0.365	0.456	3.792	0.426
Corticosterone	0.654	0.389	0.001	0.552	-0.042	0.740	-0.005	0.472	0.081	0.271
18-hydroxycorticosterone	5.250	0.618	0.027	0.401	-0.648	0.849	-0.159	0.425	1.036	0.353
Aldosterone	48.306	0.527	-0.020	0.938	17.132	0.519	-1.062	0.406	7.690	0.337
17-hydroxypregnenolone	12.858	0.275	0.030	0.399	3.830	0.300	-0.456	0.022	1.669	0.142
17-hydroxyprogesterone	-2.197	0.864	0.016	0.630	-0.304	0.933	-0.376	0.054	-0.517	0.753
11-deoxycortisol	-2.719	0.810	-0.014	0.678	-4.748	0.157	0.167	0.409	0.143	0.927
18-oxycortisol	111.300	0.516	0.485	0.076	3.532	0.941	-3.508	0.082	-	-
18-hydroxycortisol	6.080	0.224	0.016	0.310	1.093	0.507	-0.106	0.267	0.581	0.267
21-deoxycortisol	18.717	0.624	0.186	0.060	5.237	0.667	-0.294	0.592	-1.433	0.779
DHEA	11.098	0.215	-0.021	0.682	-0.591	0.849	-0.341	0.167	4.000	0.106
DHEA-S	24.671	0.293	<0.001	0.591	0.009	0.022	0.000	0.166	0.002	0.393
Androstendinoe	24.671	0.293	-0.006	0.936	6.793	0.341	-0.369	0.379	2.456	0.337
11-ketone-testosterone	25.040	0.506	0.049	0.659	4.383	0.705	-1.040	0.107	6.635	0.085
11b-hydroxytestosterone	38.130	0.354	0.016	0.899	5.167	0.690	-0.878	0.236	6.749	0.097

We performed a univariate linear regression analysis of blood pressure levels at admission. The results showed that 24 h urinary epinephrine, 11-deoxycortone and corticosterone had significantly influence on systolic blood pressure (Table 6). After adjustment of 24 h urinary epinephrine,corticosterone still had a significant effect on systolic blood pressure (b=6.712, P=0.025), but the effect of 11-deoxycorticosterone was no longer significant (b=180.474, P=0.252) (Table 7). Course of CS and 24 h urinary epinephrine had significant effects on diastolic blood pressure in univariate regression analysis, but not in multivariate regression analysis (course of CS: b=-0.580, P= 0.091; 24h urinary epinephrine: b= -2.260, P=0.186).

**Table 6:** Univariate linear regression analysis of blood pressure this visit as a dependent variable in PBMAH and CPA patients. LV: left ventricular, LA: left atrial, RWT: relative wall thickness, E, early wave transmitral diastolic velocity; A, late-wave transmitral diastolic velocity; e’, early diastolic velocity of septal and lateral myocardial portions at tissue-Doppler imaging. BMI: body mass index. CS: Cushing’s syndrome, CAD: coronary artery disease. ACEI: angiotensin-convert enzyme inhibitors, ARB: angiotensin receptor blockers. CCB: calcium channel blockers. TC: total cholesterol, TG: triglyceride, LDL-C: low-density lipoprotein cholesterol, HDL-C: high-densitylipoprotein cholesterol. FBG: fasting blood glucose, BG: blood glucose, HOMA: homeostasis model assessment. FT3: Free triiodothyronine, FT4: Free thyroxine, TSH: thyroid stimulating hormone. ACR: albumin-to-creatinine ratio. T 25OHD: total 25-hydroxyvitamin D, PTH: parathyroid hormone. GH: growth factor, IGF1: insulin-like growth factor 1. UFC: Urinary free cortisol, LDDST: Low-dose dexamethasone suppression test, HDDST: High-dose dexamethasone suppression test, HDST 24H UFC not included due to the small amount of data. DHEA: dehydroepiandrosterone, DHEA-S: dehydroepiandrosterone sulfate.

Variables	Systolic blood pressure		Diastolic blood pressure	
	b	P value	b	P value
Gender	-4.882	0.466	-3.364	0.563
Age	0.047	0.833	-0.173	0.367
BMI	0.631	0.452	-0.095	0.901
Waistline	0.598	0.082	0.382	0.207
Smoking	-3.361	0.383	-2.278	0.497
Drinking	-0.511	0.918	0.541	0.901
Course of CS	-0.415	0.187	-0.532	0.048
Duration of hypertension	-0.393	0.188	-0.444	0.084
Number of antihypertensive drugs	3.250	0.149	-0.078	0.969
ACEI/ARB	8.417	0.256	0.125	0.985
blockers	9.050	0.221	-2.092	0.747
CCB	1.889	0.757	-2.800	0.597
Diuretic (antisterone not included)	20.876	0.058	11.552	0.234
Antisterone	1.559	0.875	3.603	0.676
blockers	6.762	0.548	0.695	0.944
Combined with dyslipidemia	-15.242	0.122	-7.303	0.392
TC	1.272	0.508	0.711	0.666
TG	1.390	0.610	-1.154	0.620
LDL-C	1.425	0.573	1.753	0.416
HDL-C	3.751	0.668	-0.132	0.986
Combined with pathoglycemia	3.814	0.293	-1.865	0.556
Duration of pathoglycemia	-0.452	0.339	-0.784	0.051
FBG	0.611	0.684	-0.866	0.498
2h BG	1.091	0.192	-0.387	0.586
Fasting insulin	-0.008	0.992	0.207	0.767
2h insulin	-0.013	0.743	-0.015	0.680
Glycosylated hemoglobin	3.748	0.371	-0.589	0.872
HOMA-index	-1.002	0.674	-1.232	0.569
FT3	0.559	0.923	0.295	0.954
FT4	-1.117	0.947	3.050	0.838
TSH	7.419	0.100	7.607	0.054
ACR	0.122	0.065	0.097	0.129
Creatinine	0.122	0.562	0.134	0.453
Uric acid	0.025	0.264	0.019	0.309
GH	-2.246	0.667	-2.632	0.596
IGF1	-0.049	0.358	-0.038	0.458
T25OHD	0.212	0.511	0.414	0.127

**Table 7:** Multivariate linear regression analysis of systolic blood pressure as dependent variable in PBMAH and CPA patients.

Variables	b	P value	Variables	b	P value
24 h urinary epinephrine	-2.127	0.331	24 h urinary epinephrine	-2.042	0.181
11-deoxycortone	180.474	0.252	corticosterone	6.712	0.025

## Discussion

Generally, the incidence of CS was at least three times in women than that in men and more common in the 40–60 age group although CS could occur in any age [1] [2] [3] [18]. In sporadic PBMAH cases females were just slightly more than males, and the gender ratio was balanced among PBMAH patients with established genetic causes [17]. The median age of diagnosis for PBMAH was 55 years [17] [19]. Among the 17 PBMAH cases included in this study, the male to female ratio was 11:6 showing a higher proportion of males, but the mean age was similar to the reported literatures (55.76 years). Another study, also from Peking Union Medical College Hospital which included 102 PBMAH patients also showed a higher male ratio (male to female ratio: 61:41) [16]. In this study, the median duration from mild symptoms to diagnosis was 13 years in PBMAH cases, which was significantly longer than 1.58 years in CPA cases. The long course of disease may be due to the slow progression of PBMAH, which results in inconspicuous or mild clinical manifestations and difficult early diagnosis. It was reported that there was an average of 7.8 years between the onset of mild symptoms and diagnosis [20].

Plasma adrenal steroid levels were measured by LC-MS/MS in our study. Our results showed that PBMAH patients had higher levels of 17-hydroxyprogesterone and 17-hydroxyprogesterone, bothprecursors of cortisol synthesis that are regulated by ACTH. This may be related to lower cortisol levels and weaker inhibition of ACTH in the PBMAH group. 11-deoxycorticosterone, Corticosterone, 18-hydroxycorticosterone, and 18-hydroxycortisol, while no statistically different, showed a higher trend in PBMAH group, but 11-deoxycortisol showed a lower trend. Hannah-Shmouni F et al. [15] showed that PBMAH patients (22 females and 14 males) had elevated plasma levels of 11-deoxycortisol, corticosterone, 11-deoxycorticosterone, 18-hydroxycortisol, and aldosterone, but decreased plasma levels of progesterone, dehydroepiandrosterone and dehydroepiandrosteronesulfate compared with age - and sex-matched controls. There were no significant differences in cortisol and 18-oxycortisol between PBMAH and the control group. In particular, compared with other adrenogenic CS (16 cases of CPA, 3 cases of small nodular adrenal hyperplasia; 16 women, 3 men), PBMAH had higher corticosterone levels, and higher trend in 18-hydroxycortisol and aldosterone. Among PBMAH, the significant elevation of aldosterone was only seen in patients without the destructive ARMC5 variant. Further study showed that PBMAH adrenal samples containing ARMC5 nonsense mutation had lower expression of CYP11B2 than without, which may be the reason for the decreased aldosterone synthesis. In our subjects, the aldosterone concentration measured by chemiluminescence was higher in the CPA group.

In this study, 24 h UFC and plasma cortisol were lower in the PBMAH group than that in the CPA group, both at baseline and after LDDST. Although PBMAH patients had lower cortisol levels than CPA patients, the combined metabolic dysfunction appeared to be more severe. In this study, the proportion of hypertension combined with PBMAH was 100%, significantly higher than that in the CPA group (61.9%). There were no significant differences in diabetes (47.1% vs. 38.1%), but fasting glucose and HOMA-index levels were higher in the PBMAH group. In another study involving 102 PBMAH patients from PUMCH, PBMAH had higher rates of hypertension and diabetes than CPA (83.3% vs. 61.8%, 43.1% vs. 26.6%, respectively) [16].

Heart damage was also more pronounced in the PBMAH group. The results of echocardiography indicated that cardiac abnormalities in PBMAH and CPA were mainly characterized by cardiac hypertrophy and diastolic dysfunction, without decreased left ventricular systolic function. Comparing with CPA patients, PBMAH group had larger RV and LA diameters and higher LVEF and LVFS; although there was no statistical difference, LV mass and LV mass index tended to be higher.

Hypercortisolism has additional effects in heart influence. The activation of mineralocorticoid receptors (MR), which widely distributed in cardiomyocytes, cardiac fibroblasts, vascular smooth muscle cells and other cardiovascular systems, lead to oxidative stress, inflammation and myocardial interstitial fibrosis [21] [22]. MR, which has an affinity for glucocorticoids, areactivated by cortisol in hypercortisolism and cause heart damage. In addition, elevated plasma cortisol levels activate FOXO transcription factors, leading to significant increases in atrogin1 and ubiquitin levels, and further causing cardiomyocyte fibrinolysis and reduction of cardiomyocyte fiber content [23].

PBMAH patients had more severe cardiac influences under relatively lower cortisol levels. We conducted a regression analysis in the hope of identifying the variables contributing to the cardiac damages. The results of univariate regression analysis combined with previous understandings suggested that hypertension was an important factor affecting cardiac changes in. In CS patients, hypercortisolism can increase blood pressure by enhancing the action of corticosteroids, increasing the production of vasoconstricting substances, and regulating the activity of the renin-angiotensin-aldosterone system. Therefore, the severity of hypertension as a result of hypercortisolism in CS patients is often closely related to the severity of hypercortisolism. However, in our study, PBMAH patients had lower cortisol levels but more severe hypertension (higher rates and more medication requirements) than CPA patients, so we further explored whether other variables, especially adrenal steroid hormones, were involved in hypertension and further heart damages.

Further regression analysis showed that corticosterone had a significant promoting effect on hypertension. And compared with CPA patients, PBMAH patients had higher or a higher tendency of corticosterone. In addition to cortisol, corticosterone also appears to play an important role in the development of hypertension. Studies showed that plasma cortisol decreases and corticosterone levels increase significantly in hypertensive patients [24] [25]. In rats with hypertension induced by salt diet, the levels of corticosterone in the gut and serum were significantly increased [26]. External corticosterone increased blood pressure and induce hypertension in rats [27] [28]. The antihypertensive effect of verapamil on spontaneously hypertensive rats was accompanied by the decrease of corticosterone levels in plasma and adrenal [29]. Corticosterone inhibited the nitric oxide synthase mRNA [28], the production nitric oxide of which dilate blood vessels. In hypertensive rats and patients, significantly elevated corticosterone may activate MR and contribute to sympathetic excitation, sodium treatment and changes in vascular resistance [27] [28] [30] [31]. In addition, corticosterone also promoted sodium absorption by activating MR of intestinal epithelial cells [32] [33]. These enteral and parenteral effects of corticosterone work together to raise blood pressure. However, other studies showed that neither glucocorticoid receptor (GR) nor MR blockers affect hypertension induced by corticosterone [34]. The mechanism by which corticosterone raises blood pressure needs further investigation. Both MR activation and hypokalemia caused by corticosterone overdose can inhibit aldosterone synthesis and reduce circulating aldosterone levels [35], which seems to explain the lower aldosterone levels in the PBMAH patients in our study. Corticosterone also has additional adverse effects on the heart. Exogenous administration of corticosterone decreased free radical scavenging enzymes such as superoxide dismutase and catalase in the rat heart, and increased lipid peroxidation and protein carbonyl contents, oxidative stress markers [36]. Comparing with the control group, corticosteronelevels in the left ventricle of rats with cardiac hypertrophy were significantly increased [37]. Corticosterone increased the expression of atrial natriuretic peptide, protein synthesis and cell surface area in neonatal rat cardiomyocytes, and had an amplifying effect on cardiomyocyte hypertrophy induced by phenylephrine. However, these effects were blocked by GR blockers but not by MR blockers [37]. Hattori et al. [34] used exogenous corticosterone in rats which induced hypertension, left ventricular fibrosis and left ventricular diastolic dysfunction; increased cardiac oxidative stress and inflammation. The MR blocker, spirolactone, weakened corticosteroneinduced left ventricular fibrosis and left ventricular diastolic dysfunction, while GR blocker RU486 had no such effect.

Therefore, we speculate that corticosterone may play a role in promoting hypertension and further heart damage in PBMAH patients. Previous studies have shown that cortisol levels were the cause of more severe disease and heart changes in general CS patients. But our study focused on the rare but specific cause of CS, PBMAH, which shows lower cortisol levels but more severe heart changes.

## Conclusions

PBMAH is a rare type of CS, and its associated metabolic disorders are common. Cardiac hypertrophy and ventricular diastolic dysfunction were predominant in PBMAH patients, but left ventricular systolic dysfunction was rare. Compared with CPA patients, PBMAH patients had lower cortisol levels, but more severe hypertension, larger left atrium, and more impaired diastolic function. Among the steroid hormones, PBMAH had a high corticosterone level which may play a role in the development of hypertension and further heart changes. This study summarized the clinical and cardiac characteristics of PBMAH patients, reminding these patients that attention should be paid to aid in the clinical diagnosis of their more prone cardiac changes.

The number of cases included in this study was limited and larger studies are needed to elucidate the clinical and cardiac characteristics of PBMAH patients.

## Dodatak

### Funding

None.

### Author contributions

Sisi Miao, collected, cleaned and analyzed data, and wrote original draft. Lin Lu, designed the project, edited article, and supervised the study. Shengyong Si, Dandan Peng, Ya Zhong and Zhijing Li, collected and analyzed data. Zhenqiu Yu, designed the project, checked and reviewed article. All authors have approved the final version of this article.

### Acknowledgements

None.

### Conflict of interest statement

All the authors declare that they have no conflict of interest in this work.
